# Hormonal Regulation of Uterine Macrophages

**DOI:** 10.1155/1998/87527

**Published:** 1998

**Authors:** Joan S. Hunt, Lance Miller, Jeralyn Sue Platt

**Affiliations:** 1 Department of Anatomy and Cell Biology University of Kansas Medical Center 3901 Rainbow Blvd. Kansas City Kansas 66160-7400 USA; 2 Department of Pathology and Laboratory Medicine University of Kansas Medical Center 3901 Rainbow Blvd. Kansas City Kansas 66160-7400 USA

**Keywords:** Estrogen, interferon-γ, macrophage, mouse, nitric oxide, progesterone

## Abstract

Macrophages are major cellular inhabitants of cycling and pregnant mammalian uteri. Their densities and patterns of tissue distribution in this organ fluctuate in concert with levels of circulating female sex steroid hormones, estrogens and progesterone, and their production of various effector molecules also may be hormonally regulated. Hormonal control may be achieved by direct binding to receptors or by indirect pathways where hormones modulate production of various autocrine and paracrine cytokines and growth factors that then target to resident macrophages and influence their secretory profiles. In this paper, we marshall evidence supporting the concept that progesterone acts as a powerful negative regulator of these versatile cells, reducing their migration into the uterus and impairing their ability to produce potent effector molecules such as nitric oxide that could interfere with the success of pregnancy.


*Developmental Immunology*, 1998, Vol. 6, pp. 105–110
Reprints available directly from the publisher
Photocopying permitted by license only

© 1998 OPA (Overseas Publishers Association)
N.V. Published by license under the
Harwood Academic Publishers imprint,
part of the Gordon and Breach Publishing Group
Printed in Malaysia

### *Minireview*

### Hormonal Regulation of Uterine Macrophages

JOAN S. HUNT[b]*, LANCE MILLER[b] and JERALYN SUE PLATT[a]

[a]*Department of Anatomy and Cell Biology*; [b]*Department of Pathology and Laboratory Medicine University of Kansas Medical Center, 3901 Rainbow Blvd., Kansas City, Kansas 66160-7400*

*(Received 30 May 1997; In final form 30 May 1997)*

Macrophages are major cellular inhabitants of cycling and pregnant mammalian uteri. Their densities and patterns of tissue distribution in this organ fluctuate in concert with levels of circulating female sex steroid hormones, estrogens and progesterone, and their production of various effector molecules also may be hormonally regulated. Hormonal control may be achieved by direct binding to receptors or by indirect pathways where hormones modulate production of various autocrine and paracrine cytokines and growth factors that then target to resident macrophages and influence their secretory profiles. In this paper, we marshall evidence supporting the concept that progesterone acts as a powerful negative regulator of these versatile cells, reducing their migration into the uterus and impairing their ability to produce potent effector molecules such as nitric oxide that could interfere with the success of pregnancy.

*Keywords:* Estrogen, interferon-$\gamma$, macrophage, mouse, nitric oxide, progesterone

### INTRODUCTION

Although macrophages have long been recognized as major cellular inhabitants of cycling and pregnant mammalian uteri, nearly all of the studies that have established the fundamental characteristics of these remarkable cells have been reported within the last decade and a half (reviewed by Hunt, 1989; Hunt and Pollard, 1992). Macrophage densities and distributional patterns within cycling and pregnant uteri of rodents and women have been mapped; conditions influencing their migration, differentiation, and activation in the uterus have been investigated; and some information on their secretory profiles has been generated.

The studies done thus far indicate that essentially all aspects of macrophages in the uterus are governed directly or indirectly by female hormones, estrogens

*Corresponding author. Present address: Department of Anatomy and Cell Biology, University of Kansas Medical Center, 3901 Rainbow Blvd., Kansas City, KS 66160-7400.

and progesterone. Environmental programming is not novel to the uterus; in other contexts, macrophages are affected by cytokines as well as by extracellular matrix and cell-membrane components (Auger and Ross, 1992; Rutherford et al., 1993). Macrophages rapidly adapt to whatever task is at hand, and as a consequence, are phenotypically and functionally heterogenous.

In this paper, we present evidence in support of the concept that uterine macrophages programmed by female steroid hormones and the powerful cytokines they unleash develop functional profiles appropriate to fertility and pregnancy. The focus is on mice, a species where compartments of the uterus are very similar to the human and the type of placentation (hemochorial) is the same.

### MACROPHAGE RECEPTORS FOR HORMONES AND CYTOKINES

The exquisite sensitivity of macrophages to environmental signals is due to the presence of a wide range of receptors for modulators such as steroid hormones, polypeptide growth factors, cytokines, and bioactive lipids.

### Hormone Receptors

Several investigators have reported the presence of estrogen receptors on macrophages and this was recently verified in our laboratory by using reverse-transcription polymerase chain reaction (RT-PCR) (Miller et al., 1996). The mouse macrophage cell lines, RAW264.7 and J774, as well as culture-derived mouse bone-marrow macrophages contained estrogen receptor (ER) mRNA. Macrophages also exhibit abundant receptors for glucocorticoids (GR) (Auger and Ross, 1992), but it is not entirely clear whether the cells express androgen receptors (reviewed by Miller and Hunt, 1996).

Classical progesterone receptors (PR) cannot be identified on macrophages by direct binding nor can PR mRNA be identified by RT-PCR, yet macrophages respond dramatically to progesterone when exposed to comparatively high concentrations of the hormone. A series of elegant studies by Werb and coworkers showed that this unexpected phenomenon is due to cross-binding of progesterone to GR (Werb, 1978; Werb et al., 1978a, 1978b). Studies done recently in our laboratory where no additive effect over dexamethazone alone was obtained when macrophages were treated with a combination of progesterone plus dexamethazone (Miller et al., 1996) tend to support the cross-binding interpretation. Our current experiments are directed toward learning whether the same types of intracellular signaling pathways such as activation of the transcription factor inhibitor, I$\kappa$B$\alpha$ (Baeuerle and Henkel, 1994; Scheinman et al., 1995), occur in progesterone- and glucocorticoid-treated mouse macrophages.

### Other Receptors

Macrophages exhibit receptors for a wide range of other substances such as immunoglobulins, complement components, cytokines, colony-stimulating factors, peptides and neurotransmitters, iron-binding proteins such as transferrin and lactoferrin, lipoproteins and lipids, coagulants and anticoagulants, fibronectin, laminin and other adhesion molecules, sugars and lectins. These have recently been listed and discussed in detail by Auger and Ross (1992). Many of these substances have been identified in the pregnant uterus where they might influence macrophage behavior.

### HORMONAL INFLUENCES ON MACROPHAGE MIGRATION

The mouse estrus cycle is characterized by an increase in the levels of circulating estrogen as the cycle progresses from proestrus to estrus, and an increase in the levels of circulating progesterone as the cycle continues into diestrus-I and diestrus-II (McCormack and Greenwald, 1974). Estrogens are also high during the initial and final stages of mouse pregnancy, whereas progesterone dominates during the middle stages.

F4/80+ macrophages increase in the uterus during estrus and diestrus-I (Huang et al., 1995) and migrate into the uterus following implantation (reviewed by Hunt and Pollard, 1992), suggesting that estrogen or estrogen-stimulated uterine growth factors such as colony-stimulating factor-1 (CSF-1) or granulocyte-macrophage colony-stimulating factor (GM-CSF) may promote their migration. *In vitro* assays to test estrogen as a chemoattractant have not yet been reported. However, during the early stages of pregnancy, macrophages continue to arrive in the uterus even when CSF-1 and GM-CSF are absent (Hunt et al., 1993; reviewed by Hunt and Robertson, 1996). Possibly, chemoattraction is achieved by MIP-1$\alpha$, MIP-1$\beta$, RANTES, or JE, all of which are transcribed in pregnant mouse uterus (Robertson et al., in press). Specific message is present for all of these chemoattractants, but it is not yet known whether the transcripts are translated and functional proteins are produced.

The numbers of macrophages are dramatically reduced in diestrus-II and proestrus uteri, and the pregnancy-stimulated influx of macrophages appears to be halted during the middle stages of pregnancy. Because progesterone is high relative to estrogen during these stages, these observations are consistent with the idea that progesterone inhibits macrophage migration. This could be (1) a direct hormonal effect on the macrophage genes that promote migration such as receptors for certain adhesion molecules, (2) associated with reduction of uterine chemoattractants, or (3) due to enhanced expression of molecules such as *Fas* ligand that restrict migration of activated hematopoietic cells (Griffith et al., 1995). These possibilities remain to be explored experimentally.

### POTENTIAL REGULATION OF UTERINE MACROPHAGE ACTIVATION BY STEROIDAL HORMONES

Uterine macrophages are known to be in an activated state (high expression of Ia antigens) during the periimplantation period as well as during late stages of pregnancy (reviewed by Hunt and Robertson, 1996). Experiments are in progress to determine how activation may be achieved.

### Expression of Interferon-$\gamma$ Receptors

Macrophages are primarily activated by exposure to interferon-$\gamma$ (IFN-$\gamma$), and there is some evidence that female steroid hormones may facilitate this activation pathway in mouse uterine macrophages. *In situ* hybridization studies have demonstrated associations between levels of circulating female sex steroid hormones and the intensity of signals for IFN-$\gamma$ receptor mRNA ($\alpha$ chain, IFN-$\gamma$R) in uterine macrophages. Signals are elevated in diestrus relative to other stages of the cycle, are readily detectable in the periimplantation period, and remain high in uterine macrophages throughout pregnancy (Chen et al., 1994).

Presumably, increased expression of IFN-$\gamma$R would enhance the sensitivity of uterine macrophages to activation by IFN-$\gamma$, but this has not been demonstrated experimentally in the uterus.

### IFN-$\gamma$ in Mouse Uterus

The question then arises as to whether or not IFN-$\gamma$ is present in cycling and/or pregnant mouse uterus. Lin et al. (1993) showed that IFN-$\gamma$ is detectable in early postimplantation mouse uteroplacental units by ELISA, and limited RT-PCR analyses conducted in our laboratory indicate that the IFN-$\gamma$ gene is transcribed at both early (day 6) and late (day 18) stages of mouse pregnancy (Platt and Hunt, 1996).

The cellular source(s) of IFN-$\gamma$ are as yet unclear but might include uterine natural killer cells (uNK). In other contexts, NK cells initiate immune responses with IFN-$\gamma$ production (Sheehan and Schreiber, 1992). Alternatively, mouse placental trophoblast cells may produce IFN-$\gamma$ as is the case in the pig and human (LeFevre et al., 1990; Haynes et al., 1993). Interestingly and of potential relevance to pregnancy, the IFN-$\gamma$ gene itself is hormonally regulated; estrogen activates the IFN-$\gamma$ promoter (Fox et al., 1991).

Cause-and-effect relationships between IFN-$\gamma$ and activation of uterine macrophages via IFN-$\gamma$R have yet to be established. Although both IFN-$\gamma$- and IFN-$\gamma$R-deficient mice are available their uteri have not been examined for the presence of Ia+ macrophages. It is entirely possible that pregnancy goes forward in these knockout mice because other types of interferons (Bazer et al., 1994; Hager et al., 1994; Whaley et al., 1994) and/or tumor necrosis factor-$\alpha$ (TNF$\alpha$) (reviewed by Hunt et al., 1996) substitute for IFN-$\gamma$.

### HORMONAL REGULATION OF MACROPHAGE SECRETORY PROFILES

A limited number of observations point to regulation of uterine macrophage secretions by female hormones and/or the cytokines they control. The data collected thus far are consistent with the idea that progesterone programs uterine macrophages into a suppressive profile.

### Studies Conducted *In Vivo*

Macrophages in pregnant mouse uterus are major sites of production of the anti-inflammatory, immunosuppressive lipid, prostaglandin $E_2$ ($PGE_2$) (Tawfik et al., 1986; reviewed by Hunt, 1992). Two additional anti-inflammatory cytokines, transforming growth factor-$\beta$1 and interleukin-10 (IL-10), are produced in mouse and rat uterus (Chen et al., 1993; Lin et al., 1993) but have not been established as arising from uterine macrophages. By contrast, during most of pregnancy in rats and mice, uterine macrophages contain mRNA for the cytokine, TNF$\alpha$, but little TNF$\alpha$ protein (Hunt et al., 1993), suggesting an inhibition of production of this proinflammatory cytokine.

### Studies on Mouse Macrophage Cell Lines

Recently, we have turned to macrophage cell lines as models for studying the impact of female sex steroid hormones on macrophage secretory profiles. Nitric oxide (NO) is a major product of activated macrophages that might have a detrimental effect if produced in high amounts during pregnancy and might, therefore, be one of the proinflammatory molecules that should be restricted. It is, therefore, of considerable interest and potential importance to pregnancy that progesterone inhibits production of this potent molecule.

In macrophages, NO is produced following activation of the inducible nitric oxide synthase (iNOS) gene. We tested the ability of estrogens and progesterone singly and in combination, dexamethazone (positive control) and neutral lipids (negative controls), as well as estrogen and progesterone analogs to influence activation of the iNOS gene promoter, steady-state levels of iNOS transcripts, and production of NO (Miller and Hunt, 1996). These experiments utilized several strategies, including transient transfection of promoter constructs into RAW264.7 mouse macrophages and northern blots as well as substrate conversion assays in RAW264.7 cells, J774 cells, and cultured mouse bone-marrow macrophages.

The results of these studies showed clearly that despite the absence of detectable PR mRNA progesterone efficiently downregulates all three phases, that is, iNOS gene activation, accumulation of specific mRNA, and production of NO. This finding is consistent with a previous report from our laboratory, which demonstrated that progesterone downregulates iNOS in uterine mast cells (Huang et al., 1995), as well as the observation that progesterone inhibits the ability of macrophages to kill target cells (Feinberg et al., 1992).

### SUMMARY

The data accumulated thus far are consistent with the proposition that estrogens promote an active macrophage profile and progesterone promotes a suppressor macrophage profile. However, it is as yet uncertain whether this putative principle will hold true under changing conditions of pregnancy, when concentrations of certain macrophage-targeting cytokines and growth factors fluctuate.

It is critical also to recognize that although experimentation in this area furthers our understanding of how female steroidal hormones operate during pregnancy, the results could have broader implications. Because hormones circulate freely in the blood and macrophages are located in all tissues and organs, estrogens and progesterone are likely to have major influences on the overall immune competence in females of child-bearing age. It is, therefore, imperative to develop a full understanding of the conditions that drive tissue macrophages into the suppressive mode where reactivity against infections and tumors could be reduced.

### *Acknowledgments*

The authors thank the technical assistants, students, and postdoctoral fellows who have contributed to this work. The studies were supported in part by grants from the National Institutes of Health to J.S.H. (HD24212, HD25691), the Kansas Mental Retardation Research Center (HD02528), and the Kansas Center for Reproductive Sciences P30 Core Grant (HD33994).

### *References*

Auger M.J., and Ross J.A. (1992). The biology of the macrophage. In The Macrophage, Lewis C.E., and McGee J.O'D., Eds. (Oxford: Oxford University Press), pp. 3-74.

Baeuerle P.A., and Henkel T. (1994). Function and activation of NF-$\kappa$B in the immune system. Annu. Rev. Immunol. **12**: 141-179.

Bazer F.W., Spencer T.E., Ott T.L., and Johnson H.M. (1994). Cytokines and pregnancy recognition. In Immunobiology of Reproduction, Hunt J.S., Ed. (New York: Springer-Verlag), pp. 37-56.

Chen H.-L., Kamath R., Pace J.L., Russell S.W., and Hunt J.S. (1994). Gestation-related expression of the interferon-$\gamma$ receptor gene in mouse uterine and embryonic hematopoietic cells. J. Leukocyte Biol. **55**: 617-625.

Chen H.-L., Yelavarthi K.K., Hu X.-L., and Hunt J.S. (1993). Identification of transforming growth factor-$\beta$1 mRNA in virgin and pregnant rats by in situ hybridization. J. Reprod. Immunol. **25**: 221-233.

Feinberg B.B., Tan N.S., Walsh S.W., Brath P.C., and Gonik B. (1992). Progesterone and estradiol suppress human mononuclear cell cytotoxicity. J. Reprod. Immunol. **21**: 139-142.

Fox H.S., Bond B.L., and Parslow T.G. (1991). Estrogen regulates the IFN-$\gamma$ promoter. J. Immunol. **146**: 4362-4367.

Griffith T.S., Brunner T., Fletcher S.M., Green D.R., and Ferguson T.A. (1995). Fas ligand-induced apoptosis as a mechanism of immune privilege. Science **270**: 1189-1192.

Hager H., Aboagye-Mathiesen G., Petersen P.M., Norskov-Loritsen 388?, Juhl C.B., Villadsen J.A., Zdravkovic M., Gildberg A., Dalsgaard A.M., and Ebbesen P. (1994). Human trophoblast interferons enhance major histocompatibility complex class I antigen expression on human term trophoblast cells in culture. Placenta **15**: 709-714.

Haynes M.K., Jackson L.G., Tuan R.S., Shepley K.J., and Smith J.B. (1993). Cytokine production in first trimester chorionic villi: Detection of mRNAs and protein products in situ. Cell. Immunol. **151**: 300-308.

Huang J., Roby K.F., Pace J.L., and Hunt J.S. (1995). Localization and hormonal regulation of inducible nitric oxide synthase in cycling mouse uterus. J. Leukocyte Biol. **57**: 27-35.

Hunt J.S. (1989). Cytokine networks in the uteroplacental unit: Macrophages as pivotal regulatory cells. J. Reprod. Immunol. **16**: 1-17.

Hunt J.S. (1992). Prostaglandins, immunoregulation, and macrophage function. In Immunological Obstetrics, Coulam C., Faulk W.P., and McIntyre J.A., Eds. (New York: Norton), pp. 73-84.

Hunt J.S., Chen H.-L., and Miller L. (1996). Tumor necrosis factors: Pivotal factors in pregnancy? Biol. Reprod. **54**: 554-562.

Hunt J.S., Chen H.-L., Hu X.-L., and Pollard J.W. (1993). Normal distribution of tumor necrosis factor-$\alpha$ messenger ribonucleic acid and protein in virgin and pregnant osteopetrotic (*op/op*) mice. Biol. Reprod. **49**: 441-452.

Hunt J.S., and Pollard J.W. (1992). Macrophages in the uterus and placenta. In Current Topics in Microbiology and Immunology, Macrophages and Macrophage Activation, Gordon S., and Russell S.W., Eds. (Heidelberg: Springer-Verlag), pp. 39-63.

Hunt J.S., and Robertson S.A. (1996). Uterine macrophages and environmental programming for pregnancy success. J. Reprod. Immunol. **32**: 1-25.

LeFevre F., Martinat-Botte F., Guillomot M., Zouari M., Charley B., and La Bonnardiere C. (1990). Interferon-$\gamma$ gene and protein are spontaneously expressed by the porcine trophectoderm early in gestation. Eur. J. Immunol. **20**: 2485-2490.

Lin H., Mosmann T.R., Guilbert L., Tuntipopipat S., and Wegmann T. (1993). Synthesis of T helper 2-type cytokines at the maternal-fetal interface. J. Immunol. **151**: 4562-4573.

McCormack J.T., and Greenwald G.S. (1974). Progesterone and oestradiol-17$\beta$ concentrations in the peripheral plasma during pregnancy in the mouse. J. Endocrinol. **62**: 101-107.

Miller L., Alley E.W., Murphy W.J., Russell S.W., and Hunt J.S. (1996). Progesterone inhibits inducible nitric oxide synthase gene expression and nitric oxide production in murine macrophages. J. Leukocyte Biol. **59**: 442-450.

Miller L., and Hunt J.S. (1996). Sex steroid hormones and macrophage function. Life Sci. **59**: 1-14.

Platt J.S., and Hunt J.S. (1996). Immunoreactive interferon-$\gamma$ in cycling and pregnant mouse uterus. Biol. Reprod. **54 (Suppl. 1)**: 84.

Robertson S.A., Mau V.J. and Tramellen K.T. (In press). Cytokine-leukocyte networks and the establishment of pregnancy. Troph. Res.

Rutherford M.S., Witsell A., and Schook L.B. (1993). Mechanisms generating functionally heterogeneous macrophages: Chaos revisited. J. Leukocyte Biol. **53**: 602-618.

Scheinman R.I., Cogswell P.C., Lofquist A.K., and Baldwin A. Jr. (1995). Role of transcriptional activation of I$\kappa$B$\alpha$ in mediation of immunosuppression by glucocortocoids. Science **270**: 283-286.

Sheehan K.C.F., and Schreiber R.D. (1992). The synergy and antagonism of interferon-$\gamma$ and TNF. In Tumor Necrosis Factors, Beutler B., Ed. (New York: Raven Press), pp. 145-178.

Tawfik O.W., Hunt J.S., and Wood G.W. (1986). Implication of prostaglandin E2 in soluble factor-mediated immune suppression by murine decidual cells. Amer. J. Reprod. Immunol. Microbiol. **12**: 111-117.

Werb Z. (1978). Biochemical actions of glucocorticoids on macrophages in culture. J. Exp. Med. **147**: 1695-1712.

Werb Z., Foley R., and Munck A. (1978a). Interaction of glucocorticoids with macrophages. J. Exp. Med. **147**: 1684-1694.

Werb Z., Foley R., and Munck A. (1978b). Glucocorticoid receptors and glucocorticoid-sensitive secretion of neutral proteinases in a macrophage line. J. Immunol. **121**: 115-121.

Whaley A.E., Reddy Meka C.S., Hunt J.S., and Imakawa K. (1994). Identification and cellular localization of unique interferon mRNA from human placenta. J. Biol. Chem. **269**: 10864-10868.

